# Longitudinal overview of symptomatic and immunosuppressive drugs in LEMS

**DOI:** 10.1177/22143602261477475

**Published:** 2026-08-17

**Authors:** Wisse R. Bakker, Linda Remijn-Nelissen, Teun van Gelder, Romina Shahini, Jan J. G. M. Verschuuren, Martijn R. Tannemaat

**Affiliations:** 1 Department of Clinical Pharmacy & Toxicology, Leiden University Medical Centre, Leiden, The Netherlands; 2 Department of Neurology, Leiden University Medical Centre, Leiden, The Netherlands

**Keywords:** lamber-eaton myasthenic syndrome, amifampridine, immunosuppressive treatment, neuromuscular disease, longitudinal treatment patterns

## Abstract

Lambert-Eaton myasthenic syndrome (LEMS) is a rare autoimmune neuromuscular junction disorder characterized by muscle weakness and autonomic dysfunction. While amifampridine is the established first-line symptomatic treatment, longitudinal data regarding the evolution of therapeutic regimens over time and the use of immunosuppressive therapies remain limited. This retrospective study analyzed 70 LEMS patients treated at the Leiden University Medical Center (LUMC) between 1992 and 2023. Treatment was evaluated and a four-point “leg score” was developed and used to retrospectively quantify functional impairment and response to amifampridine. Sixty-six percent of the cohort was female, the median age of diagnosis was 54 years and non-tumor LEMS was more prevalent (77%). All patients used amifampridine at some point, and 91% initiated it within six months of diagnosis. Amifampridine improved leg scores in 57% of patients. Although 58% of patients reported side effects (mostly paresthesia), discontinuation was rare (7%). Pyridostigmine was used in 81% of patients. Immunosuppressive drug use—predominantly prednisolone and azathioprine —increased over time, reaching 44% after five years. Conversely, azathioprine, prednisolone and pyridostigmine were discontinued by 50%, 38% and 32% of patients, respectively, often due to adverse effects. In conclusion, amifampridine provides significant clinical benefits and remains the cornerstone of LEMS therapy. Immunomodulatory therapies were introduced gradually and their use increased over time. Compared with autoimmune myasthenia gravis, immunomodulatory therapy was used less frequently during the early disease course of LEMS.

## Introduction

Lambert-Eaton myasthenic syndrome (LEMS) is an autoimmune disorder affecting the neuromuscular junction. This rare disease has a point prevalence ranging from 2.3 to 3.5 per million individuals.^1–3^ Approximately 60% of LEMS patients have an associated malignancy, most commonly small cell lung cancer (SCLC).^1^ In approximately 90% of cases, autoantibodies against P/Q-type voltage-gated calcium channels (VGCC) are detectable.^4,5^ These autoantibodies inhibit the release of acetylcholine at the neuromuscular junction,^6^ resulting in muscle weakness and autonomic dysfunction.^1^ Proximal leg weakness is the most common motor symptom in the early phase of LEMS.^7^

Therapeutic approaches for LEMS are categorized into symptomatic and immune-directed treatments.^8^ Since 1983, amifampridine has been the symptomatic treatment of choice and is the only drug currently approved by both the FDA and EMA for LEMS treatment, although the off-label use of immune suppressive drugs is common.^9^ Multiple clinical trials have emphasized its role as the first-line symptomatic therapy.^10–12^ In the Netherlands, two types of amifampridine are available: the market approved product Firdapse, an immediate release (IR) tablet, and a compounded product, a modified release (MR) tablet produced by the pharmacy of Leiden University Medical Center (LUMC).^13^

Current knowledge on the use of immunosuppressive therapies in patients with LEMS is largely derived from studies conducted in myasthenia gravis (MG). Although there is some information on the use of other pharmacological treatments in LEMS,^4,7,13^ detailed data on the longitudinal evolution of therapeutic regimens in LEMS is lacking.

The LUMC is a nationwide referral center for LEMS in the Netherlands, and as a result the majority of LEMS patients in the Netherlands are or have been treated by a neurologist at the LUMC at some point during their disease course.^13^ We therefore conducted a retrospective study to investigate longitudinal treatment patterns of the use of LEMS specific therapies.

## Methods

We performed a retrospective study of all patients with a diagnosis of LEMS seen by a neurologist at Leiden University Medical Center, a tertiary center for the treatment of neuromuscular junction disorders, with clinical follow-up recorded in the electronic medical record up to December 31, 2023. Diagnosis was based on characteristic clinical features, supported by either the presence of antibodies to VGCC or abnormal decrement and ≥60% increment on repetitive nerve stimulation.^4^ Data of all included patients were retrieved from the electronic medical record (EMR). The study protocol was reviewed by the institutional ethics review committee. Due to the retrospective nature of the study, it was deemed not to be subject to the Dutch Medical Research Involving Human Subjects Act (WMO) and the need for informed consent was waived.

The study had two main aims. First, we aimed to describe longitudinal treatment patterns in LEMS, specifically to understand at what points during the disease course different therapeutic strategies—symptomatic and immunosuppressive—were initiated. Second, we aimed to evaluate the effect of treatment, where possible. For immunosuppressive therapies, this was not feasible due to their typically long latency to clinical effect and the retrospective nature of our data, with inconsistent collection of quantitative outcome measures. For these reasons, systematic evaluation of treatment effect was only possible for amifampridine, which is initiated early in the disease course and has a rapid and clinically noticeable effect. This justified a more detailed analysis of this therapy.

Epidemiological and clinical data collected consisted of: date of birth, date of diagnosis, gender, VGCC antibody status, presence of small-cell lung carcinoma (SCLC), date of death, HLA genotype, DELTA-P score, and symptoms at diagnosis. For all LEMS-specific therapies, start and discontinuation dates were collected. If available, reasons for discontinuation were extracted from the EMR. If multiple reasons were recorded, the primary reason for discontinuation was reported.

As mentioned, we aimed to systematically evaluate the effect of amifampridine in a more quantitative way. In the absence of consistent formal outcome measures such as QMG scores, we devised the ‘leg score’, a semi-quantitative outcome measure describing the functional limitations of the most common symptom for LEMS patients, proximal leg weakness, and suitable to use for a retrospective analysis. In order to do this, we converted narrative, patient-reported data on proximal muscle weakness from the EMR into a graded functional score consisting of four different categories. These different categories were: no muscle weakness (0), functional impairment while walking the stairs (1), functional impairments when walking (2), and the need of a walking aid or functional impairment in activities of daily living due to leg weakness (3). This leg score was collected before the diagnosis of LEMS and after the start of amifampridine, when the dose of amifampridine was stable. The primary researcher analyzed the full dataset, while a second investigator independently reviewed 15% of the dataset to validate the interpretations.

In addition to the effect of amifampridine, we assessed whether side effects of amifampridine were documented in the EMR. We also collected data on the amifampridine dosages used. Two different formulations of amifampridine are available in the Netherlands. We therefore analyzed which formulation was used and which was preferred by the patients, defined as the formulation recorded at the latest available documentation in the electronic medical record. Additionally, we collected qualitative data on the differences in effect patients perceived between the immediate release tablet and the modified release tablet.

### Statistical analysis

Data were analyzed descriptively. Changes in leg score within patients were assessed using the Wilcoxon signed-rank test, and differences in leg score improvement between treatment groups were assessed using the Kruskal–Wallis test. Statistical analyses were performed using R software version 4.4.0.

## Results

A total number of 70 patients with LEMS were included, with a median disease duration of 6 years with the year of diagnosis ranging from 1992 to 2023. Median duration of follow-up was 58 months, varying from less than 1 months to 337 months. Patient characteristics are shown in Table 1 for the total LEMS population and for the subgroups with or without small cell lung carcinoma (SCLC). Non-tumor (NT) LEMS was more common than SCLC-LEMS (77% vs. 23%). The median age at diagnosis was 54 years for NT-LEMS and 59 years for SCLC-LEMS. Two-thirds of all patients were female. VGCC antibodies were present in 90% of patients. HLA haplotype B8+DR3+ was more common among NT LEMS compared to SCLS-LEMS, 38% versus 17%, and a DELTA-P score of ≥ 4 was more common among SCLC LEMS, 37 versus 9%, although HLA haplotype B8+DR3+ and DELTA-P scores were only available for 70% and 59% of patients respectively.

**Table 1.** Patient characteristics.

​	LEMS (total population)	LEMS (SCLC)	LEMS (NT)	Missing data
Number of patients, n (%)	70 (100)	16 (23)	54 (77)	-
Female sex, n (%)	46 (66)	12 (75)	34 (63)	-
VGCC antibodies, n (%)	63 (90)	14 (88)	49 (91)	2 (3)
Median years of diagnosis (range)	54 (13-79)	59 (49-68)	54 (13-79)	1 (1)
Median months of follow up (range)	58 (0-337)	21 (2-164)	72 (0-337)	1 (1)
HLA B8+DR3+, n (%)^a^	16/49 (33)	2/12 (17)	14/37 (38)	21 (30)
DELTA-P ≥ 4, n (%)^a^	6/41 (15)	3/8 (38)	3/33 (9)	29 (41)

^a^Percentages calculated minus the missing data in the corresponding group.

Abbreviations: VGCC, Voltage Gated Calcium Channel; SCLC, Small Cell Lung Cancer; NT, Non-tumor associated.

Throughout the disease course, all patients used amifampridine at some point, and more than 80% used pyridostigmine, shown in Table 2. Prednisolone and azathioprine were the most frequently used immunosuppressive drugs, with respectively 49% and 42% reporting to have ever used these drugs. Use of other steroid sparing immunosuppressives was not as common, with mycophenolate being the most frequent (13%), followed by ciclosporin (6%) and cyclophosphamide (1%). Twenty-one patients (30%) had used IVIG or PX; 12 had used these treatments incidentally and 9 had used them continuously. Among these 21 patients, 13 had used IVIG (of whom 7 continuously), 4 had used PX (of whom 2 continuously) and 4 had used both.

**Table 2.** Number of patients initiating and discontinuing LEMS-specific therapies with collected reasons for discontinuation of LEMS-specific therapies.

​			Reason for discontinuation^a^		
Drug	Patients, n (%)	Discontinued, n (%)	No effect	Side effects	Remission
*Symptomatic therapies*					
Amifampridine	70 (100)	5 (7)	0	1	4
Pyridostigmine	57 (81)	18 (32)	7	9	2
*Immunosuppressive therapies*					
Prednisolone	34 (49)	13 (38)	2	5	5
Azathioprine^b^	30 (42)	15 (50)	2	12	0
Mycophenolate mofetil	9 (13)	6 (67)	3	2	1
Ciclosporin^c^	4 (6)	4 (100)	1	2	0
Cyclophosphamide^b^	1 (1)	1 (100)	0	0	0
Regular IVIg/PX^d^	9	​	​	​	​

^a^Multiple reasons for discontinuation could be recorded; only the primary reason for discontinuation is shown in this table.

^b^*Reason for discontinuing therapy is unknown in one patient*.

^c^*In one patient reason for discontinuation was pregnancy*.

^d^*Reason for discontinuation not collected*.

Abbreviations: IVIg, intravenous immunoglobulin therapy; PX, plasma exchange.

Amifampridine was discontinued in a relatively small group of patients (7%), among these patients, remission was the most frequently reported reason for discontinuation (80%). Pyridostigmine was discontinued in 32% of patients, mostly due to side effects (50%) or no clinical benefit (39%). Gastrointestinal complaints were most frequently reported, 28%, as the reason for discontinuing pyridostigmine. Prednisolone was discontinued in 38% of patients, most commonly due to remission or side effects (both 39%). Azathioprine was discontinued in 50% of patients, most commonly due to side effects (80%).

**Figure 1.**
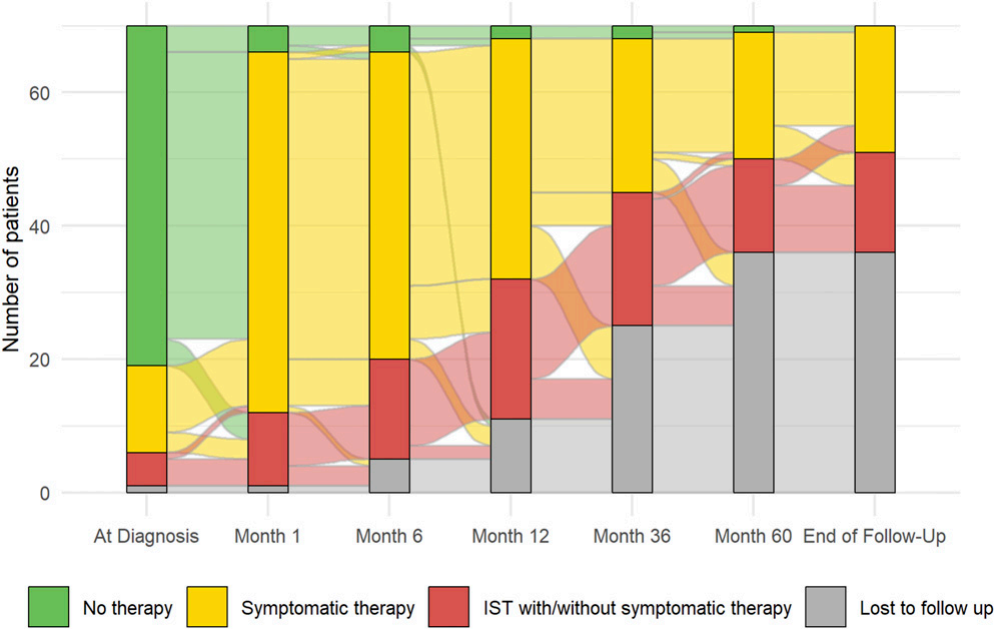
Alluvial chart illustrating longitudinal changes in LEMS therapy throughout the disease course. *Symptomatic treatment can be amifampridine and/or pyridostigmine. Immunosuppressive treatment (IST) can be prednisolone, other immunosuppressive drugs like azathioprine and intravenous immunoglobulins or plasma exchange.*

Figure 1 shows the use of different LEMS-specific drugs over the course of the disease. The majority of patients received at least one treatment one month after diagnosis (94%), with treatment use increasing as the disease progressed. The use of immunosuppressive treatment increased over time, with 15/65 (23%) patients initiating immunosuppressive treatment within the first 6 months and 14/34 (44%) patients receiving immunosuppressive treatment at 5 years. Surprisingly, 18/69 (26%) patients received symptomatic or immunosuppressive treatment before being diagnosed with LEMS.

**Figure 2.**
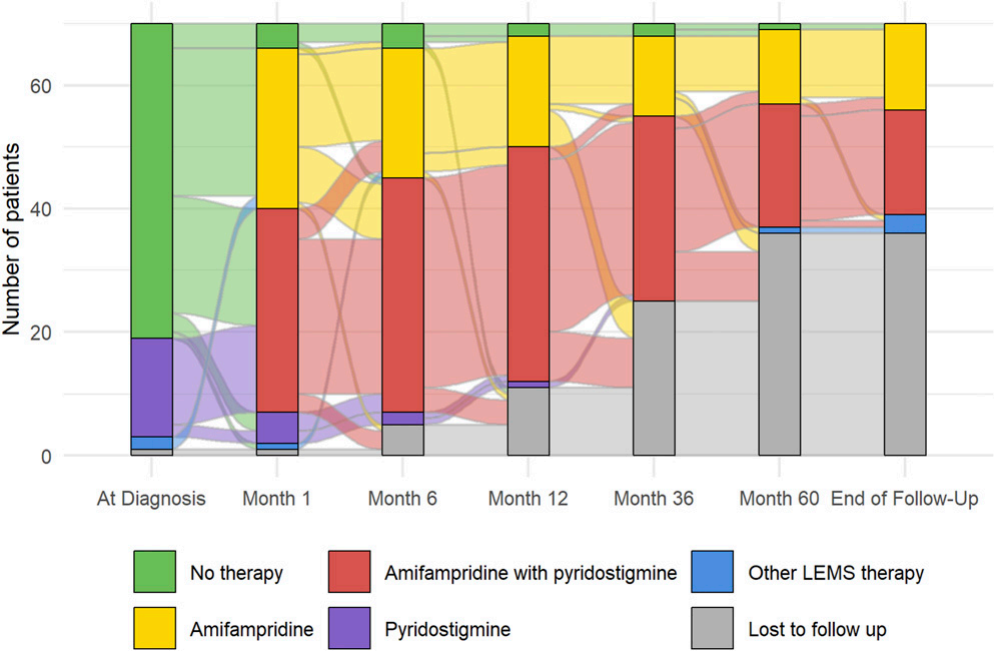
Alluvial chart illustrating longitudinal changes in symptomatic LEMS therapy over time. *Other LEMS therapy is immunosuppressive therapy or IVIG/PX for LEMS without the addition of a symptomatic drug.*

The use of symptomatic treatments over the disease course is shown in Figure 2. Within the first 6 months 59/65 (91%) patients used amifampridine, of whom 38/65 (59%) combined this treatment with pyridostigmine. The majority of patients who had used amifampridine were using the MR formulation 63/68 (93%), either alone 53/68 (78%) or in combination with the IR formulation 10/68 (15%), at the latest available documentation in the EMR. Before diagnosis, 13/69 (19%) started pyridostigmine as symptomatic treatment.

**Figure 3.**
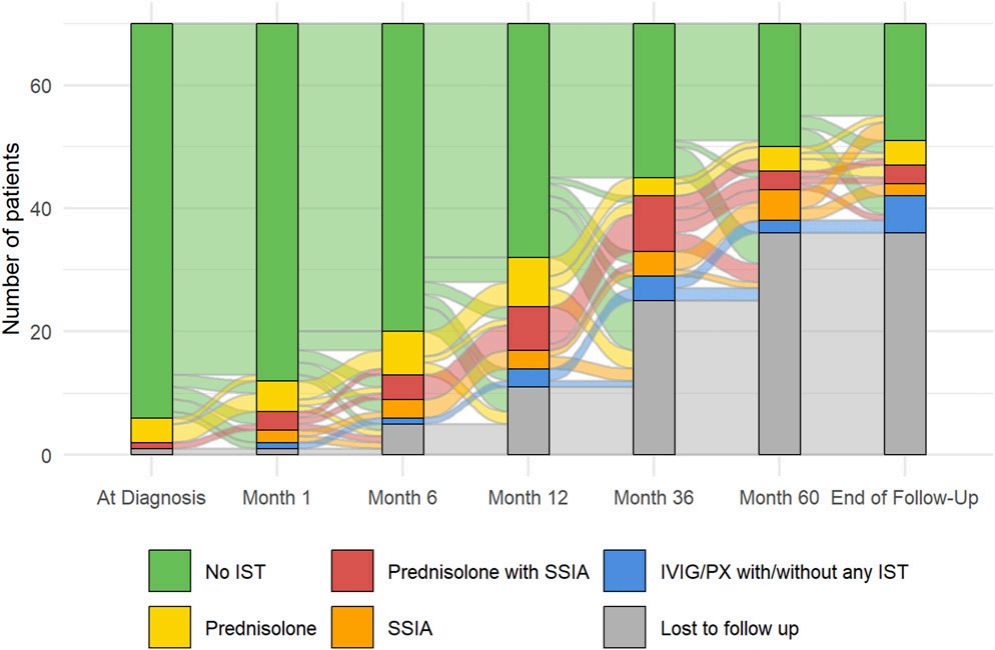
Alluvial chart illustrating the longitudinal changes in immunosuppressive LEMS therapy over time. Abbreviations: IST: Immunosuppressive, SSIA: Steroid sparing immunosuppressive agent, IVIG: Intravenous immunoglobulins, PX: Plasma exchange.

Figure 3 illustrates the use of immunosuppressive therapy throughout the disease course. At 6 months, 7/65 patients (11%) were treated with prednisolone alone and 4/65 (6%) received prednisolone in combination with a steroid-sparing immunosuppressive. At year 5, these numbers were 4/34 (12%) and 3/34 (9%), respectively. The proportion of patients using only a steroid-sparing immunosuppressive increased from 3/65 (5%) within the first 6 months to 5/34 (15%) at year 5. Chronic use of IVIG/PX was uncommon during the first 6 months of follow-up (1/65, 2%; PX only), increased to 2/34 (6%) at year 5 (1 IVIG, 1 PX), and reached 6/34 (18%) by the end of follow-up (4 IVIG, 2 PX).

**Figure 4.**
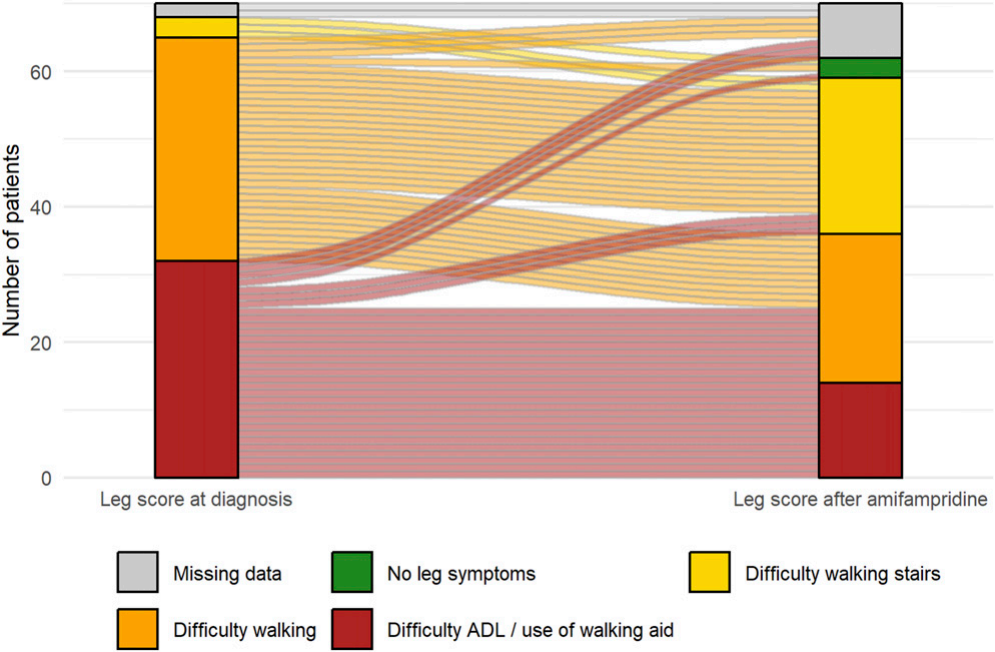
Alluvial chart depicting the change in Leg score after start with amifampridine. Abbreviations: ADL: Activities of daily living.

With regard to amifampridine treatment, both effectiveness and safety outcomes were evaluated. The effect of amifampridine on the leg score is shown in Figure 4. Following initation of amifampridine, leg scores improved in 35/62 (57%) patients. In most patients the leg score improved by 1 point (48%), while improvements of 2 or 3 points were observed in 7% and 2% of patients. Initial interrater agreement between the two assessors was high (90%). Discrepancies were limited to adjacent categories and were resolved by consensus. As shown in Figure 1, amifampridine was initiated either alone or with pyridostigmine, in addition there are patients already using pyridostigmine before initiation of amifampridine. Leg scores improved significantly after initiation of amifampridine (median change −1; Wilcoxon signed-rank test, p < 0.001). No statistically significant differences in leg score improvement were observed between treatment-naïve patients and those receiving concomitant or prior pyridostigmine or immunosuppressive therapy (Kruskal–Wallis test, p = 0.75).

Regarding safety, a total of 58% of patients reported side effects during treatment with amifampridine (Table 3), with paresthesia most frequently reported (41%), followed by cold or discolored extremities (9%). Seizure was reported in 1 patient (1%). This patient used 120 mg of amifampridine daily. After a dose reduction to 90 mg, no further convulsions were reported. One patient reported debilitating hyperhidrosis caused by amifampridine use, for which he received local treatment with botulinum toxin.

**Table 3.** Reported side effects of amifampridine.

Side effect	Documented, n (%)
Total	42 (58,6)
Parasthesia	29 (41,4)
Cold and/or discolored extremities	6 (8,6)
Nausea/epigastric discomfort	5 (7,1)
Diarrhea	4 (5,7)
Dizziness	4 (5,7)
Hypoesthesia	4 (5,7)
Headache	3 (4,3)
Muscle cramps	2 (2,9)
Constipation	1 (1,4)
Agitation	1 (1,4)
Sialorrhea	1 (1,4)
Hyperhidrosis	1 (1,4)
Epileptic convulsion	1 (1,4)

## Discussion

This retrospective study presents a longitudinal analysis of LEMS-specific therapy use over time. The cohort included fewer patients with tumor-associated LEMS compared to previous reports,^1^ likely reflecting referral bias. Patients with SCLC are less likely to be referred to the LUMC for long-term neuromuscular follow-up because oncological management often predominates their care and survival is shorter than in NT-LEMS. Although the DELTA-P score was not available for all patients, scores greater than four were more frequently observed in SCLC-associated LEMS. Conversely, the HLA haplotype B8+ DR3+ was more prevalent among patients without tumor association, consistent with previous findings and pointing at a different immunopathogenesis between the two forms of LEMS.^14^

Longitudinal data on medication use indicate that symptomatic treatment is typically initiated early and maintained throughout the disease course. Only a small proportion of patients discontinued amifampridine because of remission. However, patients in remission may have other reasons to continue symptomatic treatment, e.g., out of habit, due to perceived symptomatic relief (which may include a placebo effect), or based on the misconception that amifampridine prevents disease relapse. Therefore, a reliable estimation of total remission rates was not possible based on amifampridine use alone. Nevertheless, the continued need for long-term treatment in most patients probably highlights the burden of persistent disease in LEMS.

Notably, a subset of patients had already initiated pyridostigmine or immunosuppressive therapy at the time of LEMS diagnosis, most likely reflecting treatment for an initial diagnosis of MG prior to subsequent diagnostic reclassification. Unfortunately, data on prior diagnoses were not collected systematically, precluding reliable estimation of the rate of misdiagnosis in this cohort.

Immunosuppressive therapy is introduced more gradually and is used by approximately half of patients within the first few years. The number of patients receiving IVIG or plasma exchange increased over time. Tapering prednisolone was successful in only a few cases. Azathioprine was the most commonly used steroid-sparing immunosuppressive agent, although approximately 50% of patients discontinued its use, primarily due to adverse effects.

The observed increase in the proportion of patients receiving immunosuppressive therapy at 12 and 36 months could suggest selection bias, under the assumption that less severe cases are referred back to local neurologists. However, at 12 months, 10 patients were lost to follow-up, of whom 5 had received immunosuppressive treatment; at 36 months, 24 patients were lost, including 11 on immunosuppressive therapy. These findings indicate that the observed increase is unlikely to be explained solely by selection bias. The frequency of the use of immunosuppressive medication in LEMS is remarkably lower than that in autoimmune MG,^15^ particularly during the early years of disease. Several factors may contribute to this difference, including a more stable or milder disease course in LEMS. Another contributing factor may be the predominant lower-limb distribution of weakness in LEMS, which may result in less immediate functional impairment compared with the ocular, bulbar, or respiratory involvement that often occurs in MG, potentially influencing treatment escalation. Finally, delayed initiation of immunosuppressive therapy may partly be explained by concerns regarding an underlying malignancy at the early stages of the disease.

The retrospective design limited the application of standardized quantitative measures to evaluate treatment efficacy. To address this, a leg score was developed to assess early treatment response before and after initiation of treatment. Although this is not a validated outcome measure, the leg score allowed us to categorize the impairments as they were reported. Due to the simplicity of the scoring system, the leg score could be derived from narrative chart information at most time points from most patients. However, we acknowledge that the use of a non-validated, study-specific tool is a limitation. To minimize bias and ensure the consistency of our interpretations, a second researcher independently cross-checked a 15% subset. The 90% agreement between both reviewers indicates that the scoring system is straightforward and less prone to subjective differences in interpretation. Nevertheless, the use of a non-validated outcome measure remains a limitation of this study.

Early improvement in leg weakness was primarily associated with initiation of amifampridine. Although pyridostigmine and, less frequently, immunosuppressive therapy were initiated concurrently with amifampridine in patients with more severe disease, no statistically significant differences in leg score improvement were observed between treatment groups. In addition, patients who were already receiving pyridostigmine prior to amifampridine initiation showed a similar degree of improvement as treatment-naïve patients. Although the leg score was useful for retrospectively assessing lower-limb function, it does not encompass all aspects of clinical improvement experienced by patients with LEMS. Therefore, patient quotations were included to illustrate meaningful benefits in daily functioning that may not be reflected by changes in leg score alone.

**Quotes on the effect of amifampridine:**

,,I can stand on 1 leg again for the first time in 4 years’’

,, I can walk better, I feel fit, I can sit for longer.’’

,,After the first tablet I could walk again, I can now walk my evening round again’’

,,My morning erections have returned.’’

,,It is a world of difference; everything feels lighter.’’

,,It helps, but not enough.’’

,,My double vision has resolved.’’

In the Netherlands, amifampridine is predominantly administered as a modified-release formulation produced by the LUMC. Patients who had used both formulations reported in general less side effects and a prolonged effect with fewer fluctuations in symptom control with the modified-release formulation compared to immediate-release tablets (Firdapse). For patients who preferred Firdapse the fast initiation of effect was the most frequently reported reason. Of the 13 patients using immediate-release tablets at end of follow-up, 10 combined them with the modified-release formulation suggesting a perceived need for rapid symptom relief in addition to sustained symptom control. These observations are descriptive in nature, as the comparison was not prospectively assessed and no validated questionnaires were used. Reported adverse effects were consistent with those listed in the Summary of Product Characteristics (SMPC) for Firdapse.^16^ Of note, one patient used modified-release amifampridine in combination with pyridostigmine and azathioprine during pregnancy, resulting in two healthy neonates and one early miscarriage across three pregnancies. Only one published case report describes amifampridine use during pregnancy, which resulted in a healthy neonate.^17^ According to the SMPC, amifampridine is contraindicated during pregnancy due to limited clinical data and animal studies indicating an increased risk of stillbirth.^16^

In conclusion, this retrospective study provides a comprehensive longitudinal overview of pharmacological treatment patterns in LEMS. Amifampridine, predominantly administered as a modified-release formulation, demonstrated clear clinical benefit, although residual functional impairments remained common. Immunosuppressive and immunomodulatory therapies were introduced gradually and their use increased over time. Compared with autoimmune myasthenia gravis, immunosuppressive therapy was used less frequently during the early disease course of LEMS.

## Data Availability

The data that support the findings of this study are available from the authors upon reasonable request to the corresponding author, W.R.B. (w.r.bakker@lumc.nl), under a data sharing agreement.
